# Influences of 12-Week Physical Activity Interventions on TMS Measures of Cortical Network Inhibition and Upper Extremity Motor Performance in Older Adults—A Feasibility Study

**DOI:** 10.3389/fnagi.2017.00422

**Published:** 2018-01-04

**Authors:** Keith M. McGregor, Bruce Crosson, Kevin Mammino, Javier Omar, Paul S. García, Joe R. Nocera

**Affiliations:** ^1^VA Rehabilitation R&D Center for Visual and Neurocognitive Rehabilitation, Atlanta VA Medical Center, Decatur, GA, United States; ^2^Department of Neurology, Emory University School of Medicine, Atlanta, GA, United States; ^3^Department of Anesthesiology, Emory University School of Medicine, Atlanta, GA, United States

**Keywords:** aging, motor control, physical fitness, TMS, interhemispheric inhibition, neuroimaging

## Abstract

**Objective:** Data from previous cross-sectional studies have shown that an increased level of physical fitness is associated with improved motor dexterity across the lifespan. In addition, physical fitness is positively associated with increased laterality of cortical function during unimanual tasks; indicating that sedentary aging is associated with a loss of interhemispheric inhibition affecting motor performance. The present study employed exercise interventions in previously sedentary older adults to compare motor dexterity and measure of interhemispheric inhibition using transcranial magnetic stimulation (TMS) after the interventions.

**Methods:** Twenty-one community-dwelling, reportedly sedentary older adults were recruited, randomized and enrolled to a 12-week aerobic exercise group or a 12-week non-aerobic exercise balance condition. The aerobic condition was comprised of an interval-based cycling “spin” activity, while the non-aerobic “balance” exercise condition involved balance and stretching activities. Participants completed upper extremity dexterity batteries and estimates of VO_2_max in addition to undergoing single (ipsilateral silent period—iSP) and paired-pulse interhemispheric inhibition (ppIHI) in separate assessment sessions before and after study interventions. After each intervention during which heart rate was continuously recorded to measure exertion level (load), participants crossed over into the alternate arm of the study for an additional 12-week intervention period in an AB/BA design with no washout period.

**Results:** After the interventions, regardless of intervention order, participants in the aerobic spin condition showed higher estimated VO_2_max levels after the 12-week intervention as compared to estimated VO_2_max in the non-aerobic balance intervention. After controlling for carryover effects due to the study design, participants in the spin condition showed longer iSP duration than the balance condition. Heart rate load was more strongly correlated with silent period duration after the Spin condition than estimated VO_2_.

**Conclusions:** Aging-related changes in cortical inhibition may be influenced by 12-week physical activity interventions when assessed with the iSP. Although inhibitory signaling is mediates both ppIHI and iSP measures each TMS modality likely employs distinct inhibitory networks, potentially differentially affected by aging. Changes in inhibitory function after physical activity interventions may be associated with improved dexterity and motor control at least as evidence from this feasibility study show.

## Introduction

Aging has been shown to be associated with a loss of interhemispheric inhibition that may negatively affect unimanual motor performance of the dominant hand (McGregor et al., 2012; Fujiyama et al., 2013; Heise et al., 2013, 2014; Levin et al., 2014; see also Spirduso, 1975; Salthouse, 1996; Talelli et al., 2008). Though the motor system is relatively spared as compared to other cognitive domains such as executive function, aging is associated with decreased upper extremity function (Salthouse, 1996). While impaired inhibitory function may not reach clinical significance for diagnostic purpose of motor dysfunction, it may reveal evidence of aging-related alteration of cortical function. This loss of interhemispheric inhibition can be assessed with transcranial magnetic stimulation (TMS). One TMS measure that has shown variability due to aerobic fitness and aging is the ipsilateral silent period (iSP) (McGregor et al., 2011; Davidson and Tremblay, 2013; Coppi et al., 2014). Briefly, the iSP is a stimulation-induced diminution or cessation of oscillation in electromyography (EMG) of a contracted muscle when stimulation is given to the motor cortex ipsilateral to the muscle target. This effect is believed to be mediated by alterations in inhibitory network function (Irlbacher et al., 2007; Lenzi et al., 2007), which may be sensitive to changes in aerobic capacity (Maddock et al., 2016). Previous cross-sectional work has shown that regular aerobic exercise may be associated with changes in interhemispheric inhibition and motor dexterity in older adults (Voelcker-Rehage et al., 2010; McGregor et al., 2011, 2013). This relationship may indicate that aging related motor declines might be mitigated or even reversed by the engagement in aerobic exercise. While the effect of acute exercise has been probed with respect to sensitivity to measures from TMS (Roig et al., 2012; Singh et al., 2014; Lulic et al., 2017), we know of few studies that have assessed the longitudinal effects of a longer-term aerobic exercise program on TMS measures of inhibitory function potentially sensitive to aging and motor control (see Gomes-Osman et al., 2017).

The iSP is a complicated measurement that involves a number of cortical and descending spinal inhibitory connections. The silent period onset is typically ~38 ms after stimulation and can last anywhere from 10 to 70 ms depending on stimulation and level of muscle contraction (Giovannelli et al., 2009; Petitjean and Ko, 2013; Fleming and Newham, 2017; Kuo et al., 2017). The ipsilateral inhibition seen in the most commonly measured muscle, the first dorsal interosseous (FDI), certainly involves primary motor cortex (M1) callosal transfer, as degradation of the corpus callosum diminishes the measure (Meyer et al., 1995; Li et al., 2013). However, it is likely that inhibitory influences of the reticulospinal and propriospinal tracts provide an additive effect to muscle quiescence (Nathan et al., 1996; Ziemann et al., 1999). In addition to cortically mediated mechanisms the cause of the silent period is also influenced by the dynamics of the alpha-motoneurons themselves (Doherty et al., 1993). Refractory periods of the muscle spindle and the reactive involvement of spinal inhibitory interneurons due to descending ipsilateral corticospinal/corticobulbar/oligospinal input likely contribute to the duration of the iSP if not in its early phase, but later in its delayed resolution to baseline (Jung and Ziemann, 2006). The complexity of the iSP may be of relevance to its possible sensitivity to aging related change. Given that it is a volitional response, in contrast to another measure of interhemispheric using paired pulse parameters, aging related alteration of the iSP may reflect a functional change in motor capacity (Coppi et al., 2014).

A neurotransmitter system with strong influence on cortical inhibition and likely even motor control is the gamma aminobutyric acid system (GABA). The aging process may be responsible for a decrease in GABA tone in the neocortex (Gao et al., 2013; but see also Mooney et al. (2017). However, this decrease may or may not be responsible for changes in motor performance in aging, as GABA receptors can change endogenous sensitivity levels over time (Rozycka and Liguz-Lecznar, 2017). The TMS literature has approached GABA receptor function for many years, particularly in light of aging (Sale and Semmler, 2005; Stagg et al., 2011; Davidson and Tremblay, 2013; Opie et al., 2015). A significant question has arisen as to the role of a particular subtype of GABA receptor (GABAb) in the mediation of interhemispheric inhibition with respect to how it is assessed using TMS. GABAb receptors have been implicated in two distinct measures of interhemispheric inhibition: the iSP (described above) and paired-pulse interhemispheric inhibition (ppIHI). The employ of ppIHI requires the use of two stimulators and reflects the diminution of a motor evoked potential (MEP) in a muscle target when a conditioning pulse to the motor cortex ipsilateral to the target hand precedes a test stimulation pulse (Ferbert et al., 1992). It is yet unknown if these measures involve the same cortical circuitry (inhibitory networks) or reflect complementary findings in the estimation of the effects of pharmacological agents (see Ziemann et al., 2015). That is, the relationship between the two measures may be isolated to the administration of GABAb agents, and may not have a direct relationship with motor behavior. The current work seeks to investigate if physical activity, which has shown been associated with differences in silent period duration in previous cross-sectional work, shows proportional effects on measures of ppIHI.

The present work describes data collected from 21 older participants (60+ years) who engaged a 12-week exercise intervention comprised of either an interval-based aerobic spin program or a non-aerobic, balance and stretching condition. We employed a crossover design to compare the effects of the activity interventions on the same participants in alternate conditions. As such, participants crossed over into the alternate exercise condition for another 12-week intervention. We sought to test motor dexterity and assessments of cortical inhibition using both ppIHI and iSP paradigms that may be associated with improved motor function after increased aerobic activity. Based on our previous cross-sectional data, we hypothesized that participants completing the aerobic spin protocol would show improved upper extremity motor dexterity and increased levels of interhemispheric inhibition. We further hypothesized that changes in iSP after the intervention would indicate greater levels of interhemispheric inhibition as compared to ppIHI potentially due to the volitional and physiologically complex origin of the iSP.

## Methods

### Participants

In this 24-week randomized controlled crossover trial (RCT: NCT01787292), participants were randomized and divided into an aerobic, spin cycling exercise group (Spin) or a non-aerobic balance training group (Balance) to equalize contact and monitoring. Each intervention lasted 12-weeks (Arm 1), after which, the participant crossed over into the alternate arm (Arm 2) of the study for an additional, 12-week intervention (e.g., Arm 1, Balance–Arm 2, Spin). The crossover was an AB/BA uniform-within-sequences design with a limited washout period (~1 week). Data from both arms of the intervention are presented in this report.

Study personnel explained the purpose, potential risks of the experiment and completed the informed consent process with each participant following protocols approved by the Emory University's Institutional Review Board (IRB00059193) in compliance with the Helsinki Declaration. All participants gave written informed consent filed with both the Atlanta VA Research and Development Office and Emory University's IRB.

This report includes 21 participants that were recruited from a volunteer database, which included elderly individuals (60 years and over). An additional four older participants enrolled in the study, but chose to withdraw prior to completing the first arm of the intervention. To meet inclusion criteria participants had to (1) be between of 60 and 85 years of age, (2) report being sedentary, defined as not engaging in structured physical activity and/or not accumulating 30 min or more of moderate to strenuous weekly physical activity, assessed with a modified Godin Leisure Time Exercise Questionnaire—LTEQ (Godin and Shephard, 1997), (3) have no history of depression, neurological disease, including Parkinson's disease, Alzheimer's disease, multiple sclerosis or stroke, (4) report being right handed (using the Edinburgh handedness inventory Oldfield, 1971), (5) report being a native English speaker, and (6) obtain primary care physician's approval for study participation. Exclusion criteria included (1) conditions that would contraindicate TMS (e.g., seizure, stroke, tremor, etc.), (2) failure to provide informed consent, (3) hospitalization within the past 6 months, (4) uncontrolled hypertension or diabetes (reported non-compliance with prescribed management program), (5) inability to walk 400 m, and (6) significant cognitive executive impairment, defined as a score on the Montreal Cognitive Assessment (MoCA) of <24, (7) having a TMS measurement of lowest motor threshold (LMT) >66% of maximum stimulator output (MSO) (as stimulation for the paradigm was set to 150% of LMT). Due to the high incidence of prescription of hypertension medications in sedentary older adults (*n* = 12, six per group), we did not exclude individuals on these medications.

During intervention sessions, all participants wore a Polar FT7 chest strap heart rate monitor with paired monitor/wristwatch. Heart rate was taken from each participant every 2–3 min during the sessions and logged on a data sheet. On infrequent occasions (<2% of HR acquisitions), the chest strap monitor would fail to synchronize with the watch during the intervention session. In such instances, we interpolated the heart rate data from adjacent recordings within each session provided they were within reasonable ranges to each other (±~5–10 bpm). If a heart rate monitor failed to synchronize at study outset (a problem with older adults with lower resting galvanic skin responses) we would use a battery-powered pulse oximeter or an Apple Watch (Cupertino, CA) to measure heart rate at the above described intervals. For both interventions, we recorded attendance, attrition, and heart rate. All participants completed the 36 assigned sessions for each intervention though we had to accommodate more absences for individuals in the balance condition.

### Aerobic “spin” intervention protocol

Consistent with our previous study (Nocera et al., 2015), the group exercise intervention began with 20 min of Spin aerobic exercise three times a week for 12 weeks on stationary exercise cycles and was led by a qualified instructor. Importantly, the time of each session progressed based on the recommendation of the instructor by 1–2 min as needed to a maximum time of 45 min per session. Heart rate reserve was assessed using the Karvonen method (220 bpm – age = maximum heart rate; heart rate reserve [HRR] = maximum heart rate – resting heart rate). Exercise intensity began at low levels (50% of HRR) and increased by 5% every week (as deemed appropriate by the instructor) to a target maximum of 75% HRR. Participants wishing to exceed this capacity could do so for limited exercise intervals if they so choose. Target exercise intensities were adapted for participants on diuretics, ACE-inhibitors, beta-blockers based on recent recommendations in the literature (Diaz-Buschmann et al., 2014; Taubert et al., 2015) to produce equivalent aerobic capacity improvement as non-medicated individuals. These included the “talk-test” and relative physical exertion estimation using the Borg 6–20 difficulty scale (6 = lowest effort; 20 = maximum effort).

The Spin intervention took place in a climate controlled fitness facility. The instructor guided the participants through a light effort 5-min warm up (not included in data analysis), then a workout phase that included steady up-tempo cadences, sprints (increased rpm), and climbs (increased resistance). As such, the exercise routine employed an interval-based training approach. During the workout phase the target HRR reserve was maintained by averaging increases and decreases in intensity/HR. The goal was to maintain within a 10% offset from the HRR goal during the workout phase. Thus, participants were within target HRR on average across the session despite the intervals of increased and decreased workload. All participants wore HR monitors throughout each session and were instructed to attain their respective HR target range at 5-min intervals. Staff members also monitored and tracked the HR to ensure adequate intensity throughout each session. Brief weekly meetings in which each participant's HR was reviewed served as a way to encourage those with lower attendance or HR measurements to improve their performance for the next week.

### Balance/light strength training intervention protocol

The main purpose of the Balance and strength training group was to have participants engage in non-aerobic physical activities that may help reduce fall risk. Participants in the balance group were equalized to the Spin group with regards to contact and monitoring frequency. As such they reported to the same facility with the same interventionists; however, instead of progressive aerobic exercise they participated in group balance, stretching and light muscle toning exercises. Beginning at the outset of the intervention, a baseline balance assessment was taken for each individual to titrate task difficulty depending on intake stability risk. This was formally measured using the short physical performance battery (SPPB), which is a measure consisting of a top score of 12 (scores lower than 10 indicate moderate fall risk). All participants in this study had a score of 11 or greater, indicating low fall risk from the SPPB. Participants began the intervention by practicing balance exercises on foam pads using a chair for support (if necessary). Balance exercises included single leg stand, dual-task (counting backwards) and eyes closed conditions lasting about a total of 10 min. Participants increased difficulty when able to perform the balance session without use of the support chair. In place of foam pads, participants stood on less-stable air-filled pads as they advanced through the 12-week intervention. Participants were also challenged to learn to step on moveable friction pads (six-inch diameter “dots”) with variable positions on the floor. Instructors changed the positions of these pads as the session progressed to challenge participants to safely deviate center of mass location during foot placement in order to improve proprioception during gait. In addition, light strength training exercises included instructor-led bodyweight and resistance training using Theraband (Akron, OH) stretch bands. These exercises focused on improving postural support with an emphasis on abdominal engagement and lateral hip abduction. As above, we held brief weekly meetings to discuss progress within the program and workload.

Similar to the aerobic intervention time from the initial 20–45 min over the course of the 12-week intervention with a light 5-min warm up at the onset of each session. Additionally, heart rate was consistently monitored (also using the Polar FT7 chest strap monitors) to assess general intensity during each session and to advise participant to keep HR below aerobic levels (50% of HRR).

### Crossover and attrition

After completing the assessments within a 10-day period following the 12-week intervention, participants crossed over into the opposite arm of the study (e.g., exercise to spin). The participants then completed the second arm of the study for 12-weeks (36 sessions). We did not incorporate a full 12-week “washout” period (to potentially mitigate carryover effects) due to potential attrition of participants. We included a covariate model for carryover effects in our statistical analysis to attempt to account for the lack of washout.

Of note, we enrolled an additional four participants who completed baseline testing, but did not complete the first intervention arm choosing to withdraw from the study. Three of these participants were in the Balance arm, while one was in the Spin. Reasons for attrition were schedule conflicts or exigent family circumstances.

### Assessments

All assessments were done no more than 10 days before the start of or 10 days after the conclusion of each 12-week intervention period. In total there was: one baseline measurement and two post-intervention measurements. Assessment sessions did not exceed 2 h to alleviate participant fatigue, so testing was spread across two nearly consecutive days (1–3 days). We assessed behavioral performance and cardiovascular fitness on the first day and TMS measures on the second assessment day. In all cases but two, participants began behavioral assessments during mid-morning hours. All TMS sessions were completed during morning hours.

### Cardiovascular fitness assessment

To assess aerobic capacity, participants performed a YMCA submaximal fitness test on a Monark 828e (upright) or RC4 (recumbent) cycle ergometer (Vansbro, Sweden). This submaximal test was used to estimate the participant's maximal oxygen uptake (VO_2_max) prior to and after interventions. The selected submaximal test is much better tolerated than a maximum exertion treadmill test in the study's population (sedentary older adults). The YMCA-test uses an extrapolation method in which heart rate workload values are obtained at 2–4 points during stages of increasing resistance and extrapolated to predict workload at the estimated maximum heart rate (e.g., 220-age). VO_2_max is then calculated from the predicted maximum workload. Prior to beginning the test, the procedures were briefly explained and participants completed a 2-min warm-up consisting of pedaling without load so that they could adapt to the ergometer for the first minute and then pedaling with a 0.5 kg.m load during the second minute. The YMCA submax test has an *R* = 0.86 with VO_2_max and a SEE = 10% of the predicted VO_2_max (Beekley et al., 2004).

### Heart rate workload assessment

As a submaximal exercise estimate may be limited in determining the effectiveness of a physical activity intervention in a smaller sample size, we additionally calculated the average intrasession heart rate as compared to the target goal of 75% HRR. This was done during training intervals physical exertion starting in the sixth week of the spin program where participant HR target zone meet this criteria. Intervals in this zone (75% HRR) increased in frequency as training progressed up to six intervals per session in the final 3 days of the program. To analyze these data, we scaled the HR-values by 75% of HRR (6–10 assessments per session × 16–19 sessions) per participant (with the previously denoted adjustments for BP medications). As such, we divided each HR assessment by 75% of adjusted participant HRR and averaged each assessment across sessions within each participant. For example, if a given participants achieved a HR average of 114 bpm for work intervals their 75% HRR target value was 130 bpm, the score would be 0.87. We completed this HR_load_ analysis for all participant sessions and interventions. For the Balance group HR_load_ assessment, we chose the 75% HRR time blocks in mirror of their Spin intervention (*n* = 6–10 per session × 16–19 sessions). We acknowledge that this estimate may ignore gradual improvement in the Spin intervention as it relies on a single fixed resting HR for baseline.

### TMS

#### EMG

Electromyography (EMG) was taken from the FDI muscle on both hands using Ag-Ag Cl electrodes using BrainSight (BrainSight 2, Rogue Research) EMG pods. EMG is continuously acquired and stimulator driven TTL triggers a 150 ms acquisition window post TTL with 50 ms of pre-trigger baseline. A LabJack U3-LV analog to digital converter acquired amplified EMG traces with a 12-bit dual-channel analog input sampled at 3 kHz. These data were bandpass filtered from 10 to 10,000 Hz. Muscle activation was monitored with oscilloscope software package integrated into a BrainSight 2 neuronavigated positioning system. Motor evoked potential and other EMG data was exported for statistical analysis using ADInstruments LabChart. A MagVenture X100 magnetic stimulator (MagVenture, Alpharetta, GA) and a MagVenture B-60 60 cm butterfly coil were used to stimulate the left primary motor cortex during the initial mapping procedure. Maximum stimulator output (MSO) for this model is 2.2 tesla. All stimulations were biphasic and stimulation and recording devices were synchronized using TTL pulses. The coil was placed tangential to the scalp with the handle pointing backwards and 45° away from the midline for stimulation. The scalp site corresponding to the lowest stimulator output sufficient to generate a magnetic evoked potential of at least 50 mV in six out of 10 trials was defined as the area of resting motor threshold (RMT), also known as the “hotspot.” This was the site that was stimulated for the TMS assessments. It is worthy of note that this threshold determination is different from the currently accepted standard employ of a stimulus response curve analysis for measuring cortical excitability (Chang et al., 2016). We do not report on cortical excitability in the current manuscript as estimation of this according to the previous citation optimally uses more than 10 pulses.

#### Ipsilateral silent period

For iSP, the left FDI muscle was contracted via pinch grip at 25% maximal voluntary contraction (MVC) measured by pinch grip dynamometer and a stimulator output equivalent to 150% RMT was delivered to the left FDI hotspot. Recent work by Fleming and Newham (2017) has shown that these stimulation parameters are reliable in older adults. The highest acceptable RMT for participation in the current study was 66% of MSO. All participants had a RMT of 66% or less in the current study. Twenty silent period assessments were taken with brief rest breaks after every five trials to alleviate potential muscle fatigue. Participants were also instructed to request rest breaks as needed at any time during the stimulation. The iSP was determined using a longstanding visual inspection method (Garvey et al., 2001). Similar to our previous work (McGregor et al., 2011, 2013), we rectified EMG data and we determined silent period onset at background EMG activity during active pinch squeeze dropped below 20% of pinch baseline (assessed with pre-stimulus acquisition of 50 ms).

#### Paired-pulse measures

The long interhemispheric inhibition (LIHI) paired pulse procedure involved interhemispheric inhibition assessment (Ferbert et al., 1992) using a second MagVenture magnetic stimulator (R30) and a matching B-60 (60 cm butterfly coil) for stimulation of the right motor cortex. For this procedure, a conditioning TMS pulse set at 150% of RMT was applied to the right motor cortex FDI hotspot at 40 ms prior to a “test” pulse's administration of 130% of RMT to the left motor cortex. As a result of the conditioning stimulation, the test MEP's response amplitude (in the right FDI muscle) is lowered due to interhemispheric inhibitory processes (denoted as LIHI or long interval interhemispheric inhibition). The inter-trial interval was varied randomly between 4 and 6 s to reduce anticipation of the next trial and mitigate repetitive stimulation effects. Averages of MEP latencies and peak-to-peak amplitudes were calculated for each stimulation condition (baseline, IHI). Twenty baseline stimulations (test pulses without conditioning pulse) were compared with 20 conditioned LIHI stimulations for this procedure. Baseline and conditioned stimulations were interleaved to mitigate systematic cortical modulation.

#### Behavioral performance

During behavioral assessment sessions, participants performed a battery of cognitive and upper extremity motor tests. Results from the cognitive battery will be addressed in later report. Participants completed motor assessments of the dominant hand including: grip strength, the Halstead-Reitan Finger Tapping task (Reitan and Wolfson, 2013) simple reaction time, the Purdue Pegboard (peg and assembly) (Tiffin and Asher, 1948), and the Nine-Hole Pegboard task (Mathiowetz et al., 1985). Additionally, to test distal motor dexterity, participants engaged in a coin rotation task with two conditions. In the first condition (unimanual), the participant rotated a coin (U.S. quarter) 20 times as quickly as possible using the index finger, middle finger, and thumb with duration as the outcome measure. This test is used for assessment in routine neurological screening and has been shown to be diagnostic of distal motor function both in cases of suspected pathology and aging in the absence of pathology (Hanna-Pladdy et al., 2002; Hill et al., 2010). In the second condition (bimanual), the participant maintained an isometric pinch force of 20–30% of maximum voluntary force with a Jamar brand pinch grip dynamometer using a lateral grip during the rotations. Coin rotation tasks were performed with both the left and right hands. Both the hand used for coin rotation and trial condition (unimanual or bimanual task) were pseudo-randomized and counterbalanced across participants to account for potential order effects across eight runs (two left unimanual, two left bimanual, two right unimanual, two right bimanual). Accidental coin drops were noted, but excluded from consideration and the trial repeated should a drop occur. Participants were allowed 5 min of practice to acclimate to the rotation task in each task condition. Data acquisition began if the participant reported that they believed that additional practice time would not improve task performance. No participants requested additional time beyond the 5-min practice period. The difference score between the bimanual and unimanual task conditions was calculated to assess the effect of bimanual activity on rotation performance.

#### Data analysis

The current study was a uniform-within-sequences mixed-effects 2 × 2 crossover design with intervention type held as between subjects and intervention sequence (AB/BA) and period (A1B1/A1B2/A2B1/A2B2) as within subjects. A Shapiro-Wilks test was completed across measures to test data for normality. In the event of violation of normality of data, we employed non-parametric Wilcoxon rank sum tests (between subjects) or Mann-Whitney rank sum test (within subjects).

To analyze data from this design, we employed a mixed model approach (PROC MIXED in SAS) using a simple carryover (AB/BA) design with carryover adjustment for session sequence. To account for sequence carryover, we employed analysis of covariance (ANCOVA) in SAS 9.4 (Cary, NC) inclusive of sequence by period covariates against treatment effects. Least square means were adjusted for carryover from the crossover design and Tukey-Kramer mean comparisons for between subjects effects were analyzed with a Kenward-Roger degrees of freedom approximation (Kenward and Roger, 2010). Mauchly's test for sphericity was computed for session as a within subjects variable, and we applied a Greenhouse-Geisser correction to accommodate any violation. In addition, we completed a significance test for the carryover effect between sequences using a delta G^∧^2 likelihood ratio and Chi-square parameter estimation at alpha of 0.05.

We also performed a mixed-model split-plot ANOVA in JMP 12 (Cary, NC) using a restricted maximum likelihood design holding subjects as random and nested in sequence (i.e., AB/BA) to examine interaction effects of dependent variables based on sequence of presentation. This reduced model did not account for carryover covariates, but was employed to show main effects and interactions of treatments respective of change from each measurement (i.e., intervention at time A vs. intervention at time B; baseline assessment vs. intervention at time A; baseline assessment vs. intervention time at B). Comparisons of intervention effects on dependent variables are shown graphically in Bland-Altman repeated measures plots with *t*-test for intervention (Altman and Bland, 1983). In addition, we completed correlation analyses on dependent variables across sessions with output statistics reported with the non-parametric Spearman's rho due to the low sample size.

## Results

Our screening measure of physical activity (Godin LTEQ) showed a moderate relationship with estimated VO_2_ max, *p* = 0.42, *p* = 0.06. Baseline demographic data and neurophysiological correlations at the pre-session across all participants are shown in Table 1. Of note, VO_2_ was positively correlated with education and inversely correlated with BMI and RMT. Resting motor threshold was also inversely correlated with level of education across all participants in the selected sample. Interestingly, we found no significant correlation between the TMS measures at baseline. There was an effect on gender at baseline with women having slightly longer silent periods *t*_(20)_ = 1.99, *p* = 0.05 as compared to men. Baseline data for TMS and motor performance along with their correlations are shown in Tables 2–4, respectively.

**Table 1:** Baseline demographic and exercise metrics: age, education, body mass index (BMI), Handedness (as assessed by Edinburgh Handedness Inventory: Right = 1.0, Left = −1.0, assessed level of oxygen consumption during exercise (VO_2_), Modified Godin Leisure Time Exercise Questionnaire (Self-report of physical activity) and Montreal Cognitive Assessment (MoCA).

**Metric**	**Baseline (*N* = 21, 11 Female)**
Age (years)	69.05 (5.98)
Education (years)	16.23 (2.98)
BMI	29.12 (6.01)
Handedness	0.97 (0.06)
VO_2_ (ml/min/kg)	24.01 (9.29)
Godin LTEQ	11.62 (5.05)
MoCA	28.12 (2.9)

**Table 2:** Baseline transcranial magnetic stimulation measures between groups—std. dev.

**Metric**	**Baseline (*N* = 21, 11 Female)**
RMT (%MSO)	57.6 (9.55)
iSP (ms)	22.39 (4.73)
ppIHI (% baseline)	0.60 (0.19)

*No differences were evident in comparisons of resting motor threshold (RMT), ispsilateral silent period (iSP), and paired pulse interhemispheric inhibition (ppIHI)*.

**Table 3:** Baseline motor comparisons—std. dev.

**Metric**	**Baseline (*N* = 21, 11 Female)**
Purdue Peg	11.19 (1.6)
Purdue assembly	6.77 (0.72)
9-Hole Peg	24.39 (3.41)
Halstead	42.75 (7.09)
Coin rotation	
Right unimanual	16.46 (2.23)
Bimanual difference score	−2.58 (1.89)

*Tests were with dominant (right) hand unless otherwise specified. Higher score on Purdue, Halstead are better. Lower scores on 9-Hole peg and coin rotation are better. Bimanual difference score is the difference between unimanual dominant hand coin rotation and dominant coin rotation when non-dominant hand is engaged in 25% maximum voluntary contraction squeeze task*.

**Table 4:** Baseline correlations between VO_2_, demographic, and TMS measures across all participants with comparison *p*-value.

**Metric**	**VO_2__Pre**	**BMI**	**Education**	**RMT**	**iSP**	**ppIHI**
VO_2__Pre	X	X	X	X	X	X
BMI	**−0.54 (*****p*** = **0.01)**	X	X	X	X	X
Education	**0.43 (*****p*** = **0.04)**	−0.37 ns	X	X	X	X
RMT	**−0.49 (*****p*** = **0.04)**	0.36 ns	**−0.54 (*****p*** = **0.01)**	X	X	X
iSP	−0.10 ns	−0.02 ns	−0.23 ns	−0.03 ns	X	X
ppIHI	−0.11 ns	0.15 ns	0.01 ns	0.18 ns	−0.35 ns	X

*Significant correlations were evident between estimated volume of oxygen consumption (VO_2_), education, resting motor threshold (RMT). Ipsilateral silent period (iSP) and paired pulse interhemispheric inhibition (ppIHI) were not correlated with baseline demographics. Correlations use Spearman rho statistic. BOLD denotes statistical significance below p = 0.05*.

### Intervention effects—spin vs. balance

#### VO_2_ measures

**Figure 1:**
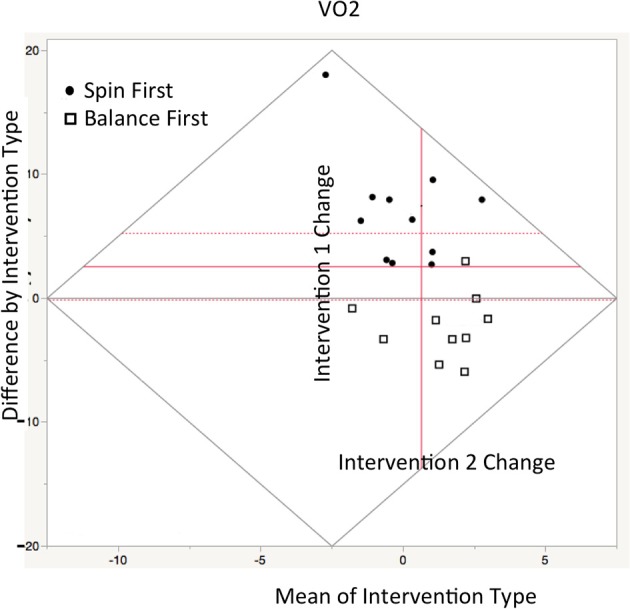
Repeated measures Bland-Altman plot of VO_2_max estimate comparisons between interventions sessions as plotted in JMP12. Ordinate axis denotes difference score between treatments, while abscissa denotes. The central axis (in red) is offset to depict the mean value between interventions A+B/2. Thus, vertical gain (from red axis) indicates greater improvement in VO_2_ in Intervention A, while rightward gain indicates greater improvement after crossover. Circles represent Spin participant in Spin first condition while boxes represent participants in balance first (Between groups comparison—means represented by dotted lines: *t* = 5.29, *p* < 0.01).

Depicted in the repeated measures Bland-Altmann plot in Figure 1, change in estimated VO_2_max was significant both accounting for carryover covariates *t*_(20)_ = 4.90, *p* < 0.001, and in the reduced model [*t*_(20)_ = 5.29, *p* < 0.001]. Interestingly, there was a significant carryover effects in the Spin First Intervention (AB/BA), $\chi_{\left(0.05,\;1\right)}^{2}$ = 6.89 (*p* < 0.03) as compared to the Balance First Intervention (BA/AB), which had no carryover effects for VO_2_ change, $\chi_{\left(0.05,\;1\right)}^{2}$ = 3.28, ns. We found no gender differences in change measures.

#### Heart rate workload

**Figure 2:**
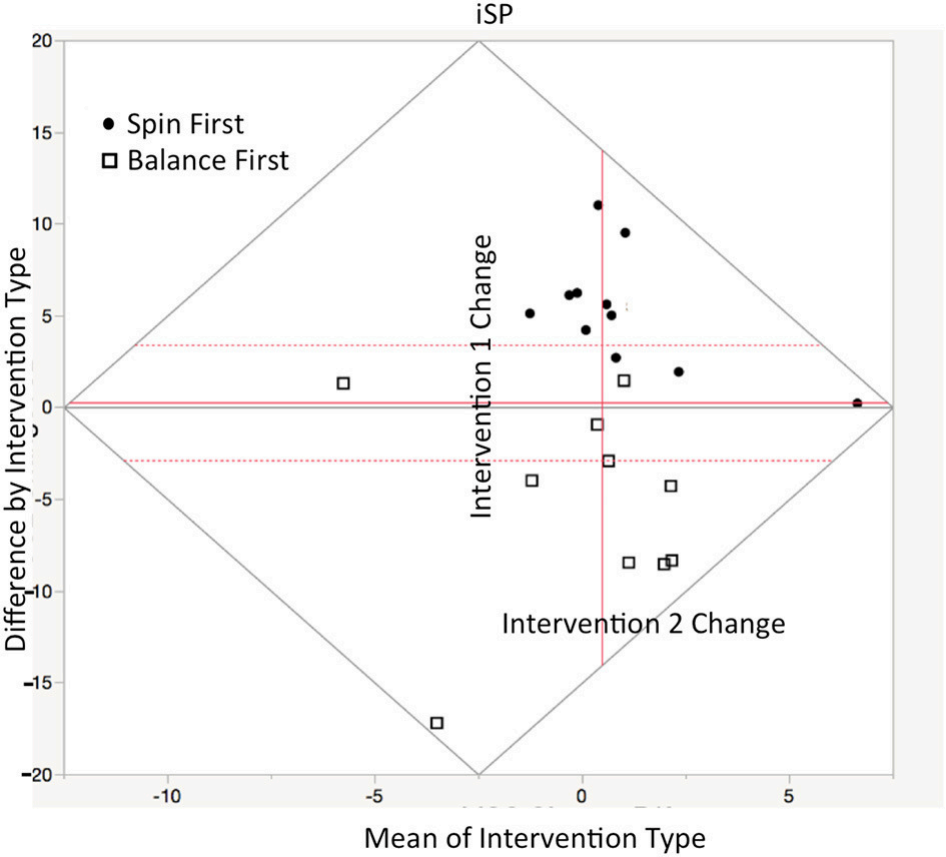
Repeated measures Bland-Altman plot of HR_load_ comparisons between interventions sessions as plotted in JMP12. Ordinate axis denotes difference score between treatments, while abscissa denotes. The central axis (in red) is offset to depict the mean value between interventions A+B/2. Thus, vertical gain (from red axis) indicates higher HR_load_ in Intervention A, while rightward gain indicates greater improvement after crossover. Circles represent Spin participants while boxes represent Balance participants (Between groups comparison—means represented by dotted lines: *t* = 2.17, *p* < 0.04).

Heart rate workload (HR_load_) was computed as a function of participants 75% heart rate reserve during intervention sessions. Heart rates in the target interval blocks were expressed as a percentage of the goal of 75% HRR. As expected, HR_load_ was higher for the Spin intervention as compared to the Balance intervention, *Z*_(20)_ = 2.27, *p* < 0.04. A significant carryover effect was evident for HR_load_ was shown $\chi_{\left(0.05,\;2\right)}^{2}$ = 8.03 (*p* < 0.01). Interestingly, using a median split within interventions at time A, we determined that individuals who performed with highest HR_load_ when performing Spin first continued to with higher HR_load_ at crossover (*n* = 5), while those performing with the lowest HR_load_ in Balance first had lower HR_load_ at crossover (*n* = 5). No gender effects were evident for heart rate data either (See Figure 2).

### TMS measures

#### Ipsilateral silent period

**Figure 3:**
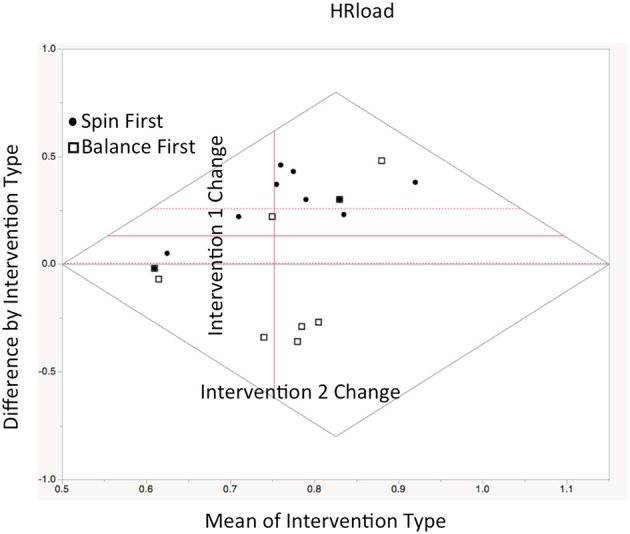
Repeated measures Bland-Altman plot of ipsilateral silent period (iSP) comparisons between interventions sessions as plotted in JMP12. Ordinate axis denotes difference measurement difference between treatments, while abscissa denotes average of both treatments. The central axis (in red) is offset to depict the mean value between interventions A+B/2. Thus, vertical gain (from red axis) indicates higher iSP in Intervention A, while rightward gain indicates greater improvement after crossover. Circles represent Spin participant in Spin first condition while boxes represent participants in balance first (Between groups comparison—means represented by dotted lines: *t* = 2.11, *p* < 0.05).

Depicted in repeated measures Bland-Altman plot in Figure 3 are change scores respective of intervention shown in the Table 5 below, there were significant effects of intervention type on change in iSP duration *t*_(20)_ = 2.11, *p* < 0.05 in the full model (inclusive of carryover), and in the reduced model, *t*_(20)_ = 4.93, *p* < 0.01. Individuals in the Spin intervention had longer iSPs than those in the balance intervention. Significant sequence carryover was present in silent period assessment for both interventions, $\chi_{\left(0.05,\;2\right)}^{2}$ = 8.89 (*p* < 0.01). We found no gender effects for change score in silent period duration, though women had a slightly higher baseline duration than men, *t*_(20)_ = 1.99, *p* = 0.05.

**Table 5:** TMS change measures after interventions.

**Metric**	**Spin**	**Balance**	***p*-value**
RMT change	−6.60 (4.1)	−7.18 (5.98)	0.54
**iSP change**	**2.22 (2.96)**	**−0.41 (2.75)**	**0.05**
ppIHI change	−0.01 (0.38)	0.04 (0.11)	0.72

*RMT, Resting motor threshold; iSP, ipsilateral silent period; IHI, paired pulse interhemispheric inhibition. iSP is measured in ms, while IHI is percentage change from baseline pulse to preconditioned pulse. BOLD denotes statistical significance below p = 0.05*.

Paired Pulse Interhemispheric Inhibition: No significant differences were denoted for ppIHI changes in the full model *t*_(20)_ = 2.13, ns, though a trend was shown in the reduced model with greater interhemispheric inhibition in the spin intervention *t*_(20)_ = 1.94, *p* = 0.07.

### Behavioral changes

Body mass index did not change respective of either intervention. Across a battery of motor indices, individuals completing the Spin Intervention improved on measures of dominant upper extremity, as compared to no change in the Balance condition. These data are shown in Table 6 and were derived from the reduced model comparison as computed by JMP12. Notably, significant differences were shown in the bimanual coin rotation task, during which the participant actively squeezes a dynamometer while rotating a coin. Participants completing the balance intervention performed the task significantly faster during the bimanual task condition as compared to little change in individuals completing the Spin intervention.

**Table 6:** Change metrics in behavioral performance comparing intervention groups—std. dev.; Purdue Peg—Higher score is better; 9-Hole pegboard and Unimanual coin rotation—lower is better.

**Metric**	**Spin**	**Balance**	***p*-value**
BMI	29.46 (6.85)	28.8 (5.51)	0.75
9-Hole Peg	**−2.3 (2.17)**	**0.95 (3.6)**	**0.02**
Purdue Peg	**1.18 (1.4)**	**−0.3 (1.15)**	**0.01**
Purdue assembly	**0.42 (1.4)**	**0.16 (1.01)**	**0.01**
Coin rotation			
Unimanual	−0.51 (3.72)	−2.21 (5.05)	0.22
Bimanual difference score	**−0.24 (2.75)**	**−3.45 (2.54)**	**0.02**

*Bimanual difference is the difference between unimanual and bimanual coin rotation tasks. Data is from reduced model as implemented in JMP12. BOLD denotes statistical significance below p = 0.05*.

### Correlations

As there do not exist ideal methods to index aging-related changes in upper extremity motor control, we performed a battery of tests. We were interested in how our TMS measures of interhemispheric inhibition related to these assessments. The data in Table 7 show significant correlations between the silent period duration and measures of distal dexterity (9-hole pegboard, Purdue, and coin rotation tasks) in aggregate after both interventions. Again, carryover considerations somewhat lessen the extensibility of these results.

**Table 7:** Relationship between TMS measures (iSP change, ppIHI % change) and motor dexterity change across all participants after both interventions regardless of order.

**Metric**	**iSP change**	**ppIHI % change**	**9-Hole Peg change**	**Purdue Peg change**	**Unimanual coin change**	**Bimanual coin difference**
iSP change	X	X	X	X	X	X
ppIHI % change	0.31 ns	X	X	X	X	X
9-Hole Peg change	**−0.80 (*p* < 0.01)**	−0.28 ns	X	X	X	X
Purdue Peg change	**0.58 (*****p*** = **0.02)**	0.34 ns	−0.21 ns	X	X	X
Unimanual coin change	**−0.51 (*p* < 0.03)**	0.32 ns	0.25 ns	−0.15 ns	X	X
Bimanual coin difference	**0.61 (*p* < 0.02)**	**0.48 (*p* < 0.05)**	**−0.56 (*p* < 0.02)**	0.43 ns	**−0.66 (*p* < 0.01**)	X

*Purdue Peg—Higher score is better; 9-Hole pegboard and Unimanual coin rotation—lower is better. Bimanual coin difference is calculated as unimanual coin rotation – bimanual coin rotation. Values are Spearman Rho calculation with df = 20. BOLD denotes statistical significance below p = 0.05*.

In addition, we performed correlations on VO_2_, HR_load_, TMS measures and motor performance to investigate relationship of the dependent measures. We performed correlations on VO_2_, HR_load_, TMS measures, and motor performance to investigate relationship of the dependent measures. Interestingly, whereas in the Balance intervention, estimates of VO_2_ were strongly inversely related to iSP (more so than HR_load_) the strongest predictor of change in iSP was HR_load_. These data are shown in Tables 8, 9 for Spin intervention and Balance intervention, respectively. We did not show any relationship between ppIHI and estimates of physical fitness/activity.

**Table 8:** Post Intervention Correlations accounting for carryover effects after Spin Intervention.

**Metric**	**VO_2_**	**HR_load_**	**iSP**	**ppIHI % change**
VO_2_	X	X	X	X
HR_load_	**0.56 (*****p*** = **0.02)**	X	X	X
iSP	**0.44 (*****p*** = **0.05)**	**0.65 (*****p*** = **0.01)**	X	X
ppIHI % change	−0.08 ns	−0.36 ns	−0.32 ns	X

*Values are Spearman Rho with alpha value in parentheses. BOLD denotes statistical significance below p = 0.05*.

**Table 9:** Post intervention correlations accounting for carryover effects after balance intervention.

**Metric**	**VO_2_**	**HR_load_**	**iSP**	**ppIHI % change**
VO_2_	X	X	X	X
HR_load_	**0.56 (*****p*** = **0.02)**	X	X	X
iSP	**−0.48 (*p* < 0.05)**	**−0.50 (*p* = 0.05)**	X	X
ppIHI % change	−0.22 ns	−0.41 ns	−0.41 ns	X

*Values are Spearman Rho with alpha significance. BOLD denotes statistical significance below p = 0.05*.

It is important to note that these data are underpowered with respect to sample size per intake group. Respective of the change in iSP, the effect size at alpha of 0.05 is 0.6. Ideally, this would require 11 participants per group. As such, we consider these data preliminary.

## Discussion

The present study demonstrates that an aerobic spin exercise intervention appears to increase the duration of the iSP in older adults and improve measures of distal upper extremity dexterity. Increased iSP duration was correlated with improved performance across multiple distal dexterity measures. Additionally, we found that the aerobic spin condition had no effect on a paired pulse measure of long interval interhemispheric inhibition (ppIHI).

Previous research has shown that engagement in regular physical activity considered aerobic in nature is associated with increased activity of inhibitory networks within the brain (McGregor et al., 2011, 2013; Nocera et al., 2015). The current work presents the first evidence that previously sedentary individuals who engage in a relatively short-term (12-week, 36 sessions) aerobic exercise program show changes in a measure of interhemispheric inhibition, the iSP previously shown to be sensitive to aging-related change (Sale and Semmler, 2005; McGregor et al., 2011; Davidson and Tremblay, 2013; Coppi et al., 2014). Moreover, a longer silent period duration was associated with improved unimanual performance on distal dexterity in our study, potentially indicating an association of cortical inhibition with motor dexterity.

### Aging-related motor performance and the ipsilateral silent period

One of the most interesting findings in the current study relates to the relationship between iSP duration and motor performance. This work has demonstrated that an aerobic spin exercise program can increase the iSP duration in concert with improving motor performance on dexterity tasks. These findings may relate to previous cross-sectional reports in our lab that found that physically active older adults had longer silent period durations than sedentary individuals in the same age cohort (McGregor et al., 2011, 2013). The question arises as to the functional relationship between the iSP and distal upper extremity dexterity. What does a silent period increase after an intervention actually infer respective of motor capability, particularly for the purposes of rehabilitation? As the iSP is complicated involving alpha motor neuronal dynamics, callosal transfer with multiple inhibitory internetworks (including spinal), and muscular control, determination of the exact mechanism driving the change is not possible in the current work. However, it is likely that cortical changes account for more of the change than changes in the periphery (muscle capacity/tone), as previous work has repeatedly shown that increased levels of exogenous stimulation alters silent period duration more so than increased motor load (Giovannelli et al., 2009; Kuo et al., 2017). Therefore, the silent period may be a reflection of an intrinsic cortical inhibitory framework that serves to regulate interhemispheric transfer.

Toward this, the relationship between the iSP and the coin rotation task is worthy of additional discussion, as it is one the few bimanual motor tasks probed in the current report (a noted limitation). We have previously reported aging-related differences in the coin rotation task when subtracting unimanual performance from bimanual performance. Younger adults complete the coin rotation task faster than sedentary older adults in either the unimanual or bimanual conditions. However, when sedentary older adults engage in the bimanual task condition (i.e., ~25% MVC pinch grip), their coin rotation speed improves. We have previously postulated that aberrant interhemispheric transfer during a unimanual task may interfere with dexterous task performance (McGregor et al., 2012). However, during a bimanual task, the engagement of the ipsilateral motor areas (to the hand performing the rotation) might act to either improve the signal-to-noise dynamics between hemispheres resulting in improved motor dexterity. Additionally, this bimanual performance effect is sensitive to differences in physical activity levels in middle-aged and older adults (McGregor et al., 2011). In the present study, our participants who completed the aerobic spin training showed improved performance on the unimanual coin rotation task. Moreover, the difference score between unimanual and bimanual conditions was lower, particularly for the non-dominant hand. Interestingly, these data were correlated with improved silent period duration. This may indicate that improved interhemispheric inhibition after the aerobic exercise in older adults could restore motor dexterity by improving signal-to-noise characteristics in the task active cortex.

### Aerobic spin intervention

The main contrast condition in the present crossover study was the type of intervention either aerobic “Spin” or Balance. As a result of our interval-based Spin program our participants, regardless of sequence of intervention, improved estimated VO_2_ as a result of increased workload. The physical performance metrics were highly correlated with both silent period duration and tests of motor dexterity. That individuals improved on motor dexterity is notable in the present study because we did not employ a manual task training component to the study, and indeed, our intervention was largely driven by the activity of the lower extremity. With respect to the mechanism of change, increased levels of brain derived neurotrophic factor (BDNF) has received dominant attention in the literature (Kleim et al., 2006; Tang et al., 2008; Schmolesky et al., 2013; Szuhany et al., 2015). Our lab only recently began to assay serum BDNF levels, but our preliminary data indicates that our aerobic Spin interval training program increases serum BDNF levels by 17% with peaks achieved 15 min post the 45-min spin session (unpublished data). BDNF is believed to promote synaptic plasticity possibly through facilitating signaling cascades after its dimers bind to its preferential receptor, TrkB (Phillips et al., 2014). As a result, multiple proteins associated with cell survival and proliferation are produced if the TrkB receptor has the beneficial Val66Val polymorphism (Kleim et al., 2006). It is unknown what benefit BDNF or other potential modulatory neurotrophins might have on either the TMS or behavioral measures employed in the current study. It is likely that increased HR workload is associated with a higher release of BDNF (Schmolesky et al., 2013) and this would predict greater motor performance and longer silent periods. However, this postulation requires additional study to vet. As such, the specific mechanism by which aerobic exercise alters cortical function remains largely unidentified with respect to both systems physiology and molecular neuroscience. Clearly, much more work is required address this critical issue.

It is important to note that the contrast condition of Balance training was not of detriment to our participants in terms of functional outcome despite a negative correlation between silent period duration and physical activity measures. Indeed, while the participants' aerobic capacity and heart rates were similar at post-assessment as compared to immediately beginning the Balance program, a crossover effect was evident in this condition. That is, participants continued to maintain gains in the currently reported metrics if they crossed over from Spin into this condition. Moreover, beyond the contrast to Spin, the Balance and light strength training condition improved core strength in participants and improved proprioception. As such, the negative correlations to motor dexterity shown in the current study may rather reflect the dominant improvement in the Spin comparison, rather than functional declines in the Balance condition. Respective of this, the Balance condition employed in the current report served as a contact control, but perhaps not an ideal control. We previously attempted to employ a wait-list control and an education-only program and washout periods to this project, but due to the study environment and recruitment dropout, we instead chose to directly enroll participants into the Spin or Balance interventions with immediate crossover. Future work will certainly employ a cleaner study design, though the results from the current, albeit non-ideal design are extremely encouraging.

### Differences between iSP and ppIHI

The change in iSP after aerobic exercise is notable insofar that it differed from an alternative measure of interhemispheric inhibition assessed with the paired-pulse LIHI stimulation, which showed no differences after the intervention and was not correlated with motor performance. This is curious and worth some exploration since both the iSP and ppIHI protocols have been reported to involve similar neurotransmitter receptor systems and are considered complementary measures of interhemispheric inhibition (Chen, 2004; Di Lazzaro et al., 2007; Wischnewski et al., 2016). In a recent study, Li et al. (2013) identified patients with callosotomy or callosal agenesis and tested iSP duration and magnitude of ppIHI. The authors reported that both ppIHI and iSP are impaired after lesion of the corpus callosum. The inhibitory effects should not be considered identical, however, as ppIHI and iSP paradigms show different changes during pharmacological manipulations (Siebner et al., 1998; McDonnell et al., 2007; Ziemann et al., 2015) and when employed immediately after or in concert with other TMS paradigms (e.g., LICI, SICI) (Udupa et al., 2010). Based on pharmacologic investigations, it has been suggested that the interhemispheric inhibition underlying both the ppIHI and iSP paradigms involve GABA Type B (metabotropic) receptor (Ziemann et al., 2015). Our results show a difference between iSP and ppIHI and therefore suggest that it is unlikely that GABAb receptor activity is the sole mechanism of this interhemispheric inhibition. Were this true, the iSP and ppIHI measures should have been directly related in the current study. Much more study is required to elucidate the neurophysiological metabolism of inhibition using TMS methodology.

There are some noted limitations with the current work. Carryover effects in the crossover design from one intervention to the other limits the extensibility of these. Future work should employ a more appropriate control condition such as an education-only arm with equivalent frequency of participant contact. As the participants in the current study do not have motor pathology, the extensibility of these findings to clinical populations may be limited. With regard to the TMS procedures, additional metrics such as cortical excitability would have been useful to report. We additionally acknowledge that the motor assessment battery was somewhat limited. Largely due to time considerations for testing, we could only administer a relatively small number of upper extremity tests in our sessions. In addition, most tests involved engagement of the dominant hand. Given the coin rotation findings, intermanual differences should be assessed with better granularity in future work. Finally, we did not track extramural activity and lifestyle habits in the current study. As such, we cannot account for variance from various unmeasured factors (i.e., overall daily activity, inflammatory biomarkers) in our statistical models, which should be tracked closely in future work.

In conclusion, we believe the current work is the first to show that a 12-week aerobic exercise intervention may affect the duration of the iSP duration in older, sedentary adults. In addition, change in silent period duration is correlated with improvements in motor dexterity. These findings are in concert with previous data collected from cross-sectional work involving middle-age and older adults of varying physical fitness levels (McGregor et al., 2011, 2013).

## Author contributions

KMM, JN, and BC: conceptualized the experiment; KMM, KM, and JN: completed data collection and handled recruitment of participants. KMM, KM, JO, and PG: analyzed the data for the work. KMM, PG, JN, and BC wrote the manuscript.

### Conflict of interest statement

The authors declare that the research was conducted in the absence of any commercial or financial relationships that could be construed as a potential conflict of interest.
